# DNA Replication Origins and Fork Progression at Mammalian Telomeres

**DOI:** 10.3390/genes8040112

**Published:** 2017-03-28

**Authors:** Mitsunori Higa, Masatoshi Fujita, Kazumasa Yoshida

**Affiliations:** Department of Cellular Biochemistry, Graduate School of Pharmaceutical Sciences, Kyushu University, 3-1-1 Maidashi, Higashi-ku, Fukuoka 812-8582, Japan; 1ps11039n.mitsunorihiga@gmail.com

**Keywords:** DNA replication, genome integrity, telomere, shelterin, G-quadruplex, RecQ-like helicase, fragile telomere, replication fork barrier, dormant origin

## Abstract

Telomeres are essential chromosomal regions that prevent critical shortening of linear chromosomes and genomic instability in eukaryotic cells. The bulk of telomeric DNA is replicated by semi-conservative DNA replication in the same way as the rest of the genome. However, recent findings revealed that replication of telomeric repeats is a potential cause of chromosomal instability, because DNA replication through telomeres is challenged by the repetitive telomeric sequences and specific structures that hamper the replication fork. In this review, we summarize current understanding of the mechanisms by which telomeres are faithfully and safely replicated in mammalian cells. Various telomere-associated proteins ensure efficient telomere replication at different steps, such as licensing of replication origins, passage of replication forks, proper fork restart after replication stress, and dissolution of post-replicative structures. In particular, shelterin proteins have central roles in the control of telomere replication. Through physical interactions, accessory proteins are recruited to maintain telomere integrity during DNA replication. Dormant replication origins and/or homology-directed repair may rescue inappropriate fork stalling or collapse that can cause defects in telomere structure and functions.

## 1. Introduction

In eukaryotic cells, protection of the ends of linear chromosomes depends on specialized nucleoprotein structures known as telomeres, which function as buffers for the shortening of linear chromosomes during each round of semi-conservative DNA replication and prevent activation of DNA damage responses, such as the ATM and ATR checkpoint signaling, classical and alternative non-homologous end joining pathways, and homologous recombination repair [1,2,3,4]. Vertebrate telomeric DNA consists of thousands of tandem 5′-TTAGGG-3′ repeats [5]. In contrast to the small telomeres of yeasts that consist of several hundred base pairs, human telomeres are typically 10–15 kb in length, and those of mice are 20–50 kb [1]. The telomeric repeat array is bound by the shelterin protein complex that is composed of telomeric repeat-binding factor 1 and 2 (TRF1 and TRF2), repressor/activator protein 1 (RAP1), TRF1-interacting nuclear protein 2 (TIN2), protection of telomeres protein 1 (POT1), and POT1- and TIN2-interacting protein TPP1 (TINT1/PTOP/PIP1) [6]. The repeat array terminates in a single-stranded 3′ protrusion of the G-rich strand (referred to as a G-overhang). The chromosome ends are stabilized by the formation of a protective loop structure, called a T-loop (telomere loop), in which the G-overhang presumably loops back and invades the double-stranded region of telomeric DNA [7,8]. Telomeres thereby prevent chromosome ends from inappropriate recognition by DNA damage signaling and repair systems [2]. In addition, several conserved features of telomeres, such as constitutive heterochromatin, G-quadruplex (G4) DNA secondary structure, and transcription of the non-coding telomeric repeat-containing RNA (TERRA), are also involved in the regulation of telomere capping and maintenance [9,10,11,12,13].

The majority of telomeric double-stranded DNA repeats are replicated in a semi-conservative manner by conventional DNA replication machinery [14]. However, characteristic features of telomeres represent intrinsic replication fork barriers that induce stalling and/or collapse of replication machinery [3,4]. Failure of telomeric DNA replication can cause genomic instability, which in turn promotes cellular transformation or senescence [15]. Here, we summarize the recent advances in our understanding of the mechanisms that support efficient DNA replication at mammalian telomeres, with a focus on the functional interactions between shelterin components and a variety of accessory proteins that enable the replication machinery to reach the chromosomal termini.

## 2. Replication Origins for the Duplication of Telomeric DNA

### 2.1. General Regulation of Eukaryotic DNA Replication; Origin Licensing and Firing

**Figure 1:**
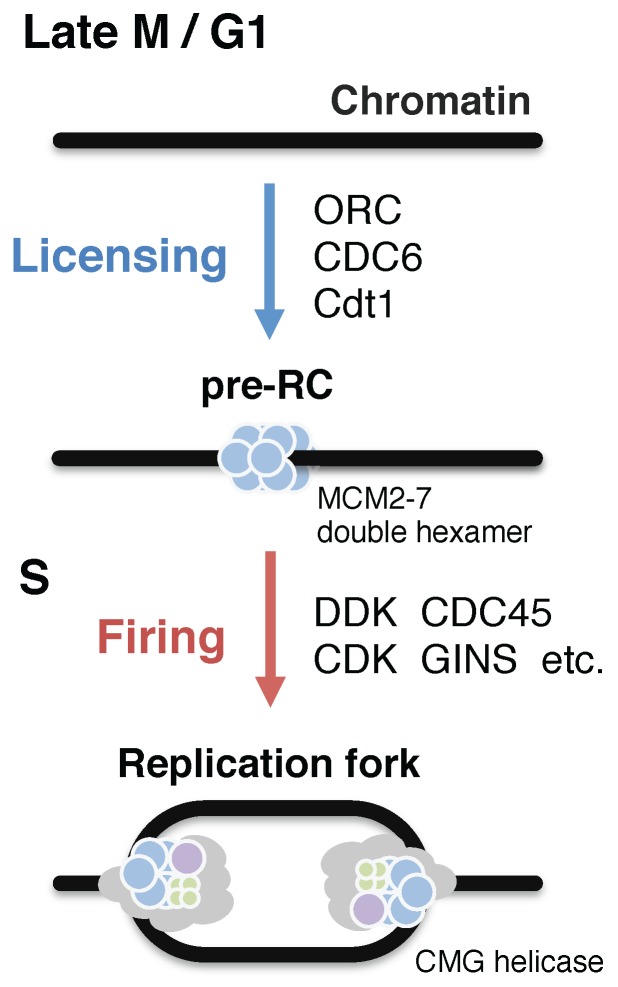
Initiation of eukaryotic DNA replication. Eukaryotic DNA replication is strictly regulated through two non-overlapping steps, origin licensing and firing. During the licensing step, which occurs from late M to G1 phases, the origin-recognition complex (ORC), and subsequently cell division cycle protein 6 (CDC6), DNA replication licensing factor Cdt1, and the MCM2–7 complex, bind to chromatin to form the pre-replication complex (pre-RC). The firing step requires S phase-specific kinases DBF4-dependent kinase (DDK) and cyclin-dependent kinase (CDK) that facilitate the loading of cell division cycle protein 45 (CDC45), the GINS complex (Sld5–Psf1–Psf2–Psf3), and several other proteins, to form the CMG (CDC45–MCM–GINS) helicase complex, which unwinds the DNA duplex, enabling DNA polymerases to initiate DNA synthesis at the replication fork. Multiple MCM2–7 double hexamers are loaded onto chromatin (not depicted in the figure). Licensed origins are sequentially activated during S phase. Some origins (called dormant origins) do not fire, are passively replicated in normal S phase, and act as backup origins upon replication stress.

The accurate DNA replication of eukaryotic genomes relies on strict temporal separation of chromatin loading of a replicative helicase (so-called origin licensing) from its activation followed by DNA synthesis (so-called origin firing) (Figure 1) [16,17]. In the late M to G1 phases, the MCM2–7 helicase complex is recruited onto chromatin in an inactive form in a process that is dependent on the origin-recognition complex (ORC), cell division cycle protein 6 (CDC6), and DNA replication licensing factor Cdt1 [18,19]. This step is also referred to as pre-replication complex (pre-RC) formation. In the subsequent S phase, DBF4-dependent kinase (DDK) and cyclin-dependent kinases (CDKs) trigger the recruitment of additional replication proteins to the origins, leading to the remodeling of inactive MCM2–7 complexes to active CMG (CDC45–MCM–GINS) replicative helicase complexes, and to the initiation of DNA synthesis at bidirectional replication forks [18,20,21]. According to a recent model, DNA polymerase α (Pol α) and primase complex initiate DNA synthesis, and Polδ and Polε continue lagging and leading DNA strand synthesis, respectively [22]. MCM2–7 loading is strictly inhibited after the onset of S phase through a number of redundant mechanisms, thereby preventing re-replication of the genome [23].

Positioning of sites for binding of ORC and MCM2–7 in the G1 phase is a key regulator of the chromosome-replication program, in which multiple replication-initiation sites (replication origins) are distributed along chromosomes [24,25,26,27,28,29,30,31]. Ideally, bidirectional replication forks should continue along a chromosome until they meet forks coming from adjacent origins, or they reach the end of the chromosome. However, replication forks often pause and collapse because they encounter obstacles, such as damaged DNA, interstrand DNA cross-links, or DNA-RNA hybrids that form R-loop structures, or because of exhaustion of dNTPs or of the single-stranded DNA (ssDNA)-binding protein RPA [15,32]. Because reloading of MCM2–7 in the S phase should not occur, so-called dormant origins (backup pre-RCs formed in G1 phase but not used in normal S phase) are reserved to complete genome replication in conditions of replication stress [15,33,34,35,36]. The DNA-replication-checkpoint pathway coordinates multiple mechanisms, including cell cycle arrest, protection and restart of stalled forks, and activation of dormant origins, to maintain genome integrity [37,38].

### 2.2. Replication Origins for Duplication of Telomeric DNA

In contrast to yeast telomeres, which are replicated in late S phase, human telomeres are duplicated throughout S phase [39,40,41,42,43]. Timing of the replication of human telomeres is specific for each chromosome arm and is dependent on subtelomeric elements, although the mechanism for this regulation is still unclear [41,42,44]. Unlike yeast cells, in which the telomeric protein Rif1 negatively regulates subtelomeric origin firing, mammalian Rif1 is not localized to telomeres and therefore may not play a role in regulation of telomeric DNA replication [45]. Single-molecule DNA-fiber analysis has enabled identification of replication origins labeled with thymidine analogs around telomeres in mouse and human cells [46,47,48]. Similar to the origin distribution in yeasts [49,50,51], origins are frequently found in mammalian subtelomeric regions. Moreover, in some cases, replication initiates within the telomeric repeats themselves. The results of nascent-strand sequencing (NS-seq) experiments also suggest that, even after normalizing for λ-exonuclease bias, human telomeric DNA is enriched in the sequences of actual firing origins [52].

Telomeres challenge the progression of replication machinery. Telomeric origins may function as a backup system that is needed to ensure completion of telomeric DNA replication. When a replication fork collapses within a telomere, additional origin activation could prevent telomere loss resulting from a large unreplicated region [15,33]. The genomic regions called common fragile sites are frequently broken upon replication stress. The chromosomal fragility is associated with the origin-poor regions of genomes [24,53,54]. It also stems from DNA secondary structures, collision with transcription of large genes, or condensed chromatin structures, all interfering with progression of replication fork. Defects in telomere replication similarly lead to chromosomal fragility [48,55,56,57,58], suggesting that origins in telomeric regions may be important for genome stability.

### 2.3. Mechanisms Promoting Pre-RC Formation on Telomeric DNA

Results from several studies demonstrate active ORC binding and pre-RC formation within TTAGGG repeats [59,60,61,62,63,64], and the shelterin component TRF2, which is essential for telomere capping, has been implicated in origin licensing through physical interaction with the largest ORC subunit, ORC1 [59,60,61,65,66]. TRF2 knockdown reduces ORC binding and pre-RC formation on telomeric DNA [60,61,63]. The TRFH (TRF homology) dimerization domain of TRF2, but not a mutant domain defective in dimerization, recruits ORC and pre-RC to chromatin [66]. This dimerization domain also interacts with proteins that are involved in telomere maintenance, such as 5′ exonuclease Apollo, structure-specific endonuclease subunit SLX4, regulator of telomere elongation helicase 1 (RTEL1), and RAP1 [67,68,69,70,71,72]. An interaction between ORC1 and the basic domain of TRF2 has also been proposed [59,60,65].

Several telomere-specific features may support ORC binding to telomeres. G4 DNA is a non-B-form DNA secondary structure constructed by parallel four-stranded guanine base pairing [73,74]. Systematic genome-wide studies have suggested that G4-motif sequences are associated with replication origins [24,75,76,77,78,79]. In vitro, human ORC1 physically interacts with G4-forming ssDNA and RNA [59,80]. Several lines of evidence support the presence of G4 DNAs at human telomeres [9,81,82,83]. The telomeric C-rich strand is transcribed from the subtelomeric region toward the telomere by RNA Polymerase II to generate TERRA [84,85]. TERRA then interacts with telomeres and is involved in heterochromatin organization and telomere maintenance [10,12,86,87]. TERRA–telomeric DNA hybrids form R-loop structures, which may result in the formation of G4 on the displaced G-rich ssDNA [87,88]. Further research is needed to determine whether these telomeric G4 structures promote ORC recruitment and origin firing.

Telomeric regions (and subtelomeric regions) are highly enriched with repressive epigenetic modifications [12,13]. Heterochromatin proteins that interact with ORC, such as heterochromatin protein 1 (HP1) and ORC-associated protein (ORCA, also known as LRWD1), might be involved in the regulation of telomeric replication origins [89]. ORCA localizes to heterochromatic sites including telomeres, and functions in the regulation of replication licensing through interactions with ORC, Cdt1, and geminin in a cell cycle-dependent manner [89,90,91,92,93]. Among repressive modifications of telomeres, the trimethylated lysine 20 of histone H4 (H4K20me3) is associated with ORC recruitment to replication origins [94,95]. The methyltransferase PR-Set7 (also known as Set8 and KMT5a) catalyzes H4K20 monomethylation, while other methyltransferases Suv4-20h1 and Suv4-20h2 are responsible for the transition from H4K20me1 to H4K20me2/3 [96,97,98]. Ectopic tethering of PR-Set7 promotes trimethylation and loading of ORC in a manner that is dependent on Suv4-20h1 [92,94]. Although the BAH (bromo-adjacent homology) domain of ORC1 preferentially interacts with a H4K20me2 peptide [92,99], ORC complexed with ORCA is thought to interact with H4K20me3 [92,93]. H4K20me3 is highly enriched at telomeres and other transcriptionally silenced regions [100,101,102], but the roles of this modification in telomeric replication remain to be established.

## 3. Shelterin and Additional Proteins that Support Telomeric DNA Replication

### 3.1. Telomeric Obstacles Against Passage of Replication Forks

**Figure 2:**
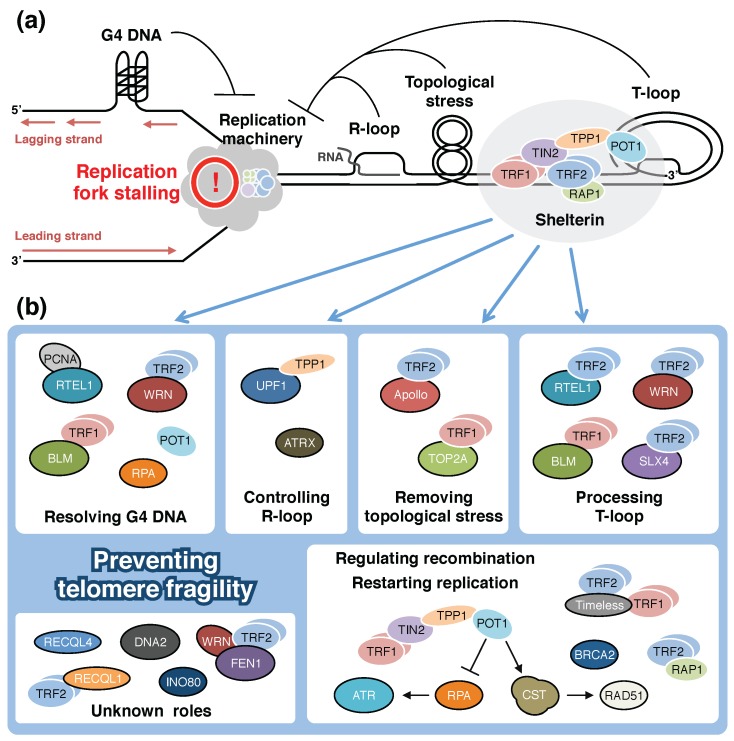
The causes of replication fork stalling and the mechanisms that overcome telomeric obstacles. (**a**) The bulk of telomeric DNA is duplicated by conventional semi-conservative DNA replication. When a telomeric replication fork progresses unidirectionally toward the chromosomal end, G-rich and C-rich strands are replicated by lagging-strand and leading-strand synthesis, respectively. The replication machinery encounters various obstacles that compromise passage of the fork through the telomere. (**b**) Telomere-specific and non-telomere-specific proteins overcome the obstacles and prevent telomere fragility during DNA replication. Components of shelterin complex have prominent roles in the recruitment of accessory factors to telomeres, while recruitment of several factors, such as RecQ-like helicase 4 (RECQL4), DNA replication helicase/nuclease 2 (DNA2), chromatin remodeling proteins INO80 and ATRX, and the DNA repair protein breast cancer 2 (BRCA2), may be independent of shelterin. The defects in many of these factors result in fragile telomere phenotype, suggesting that these obstacles naturally exist in cells and are potential causes of genomic instability. Although these factors are also involved in other telomere-maintenance mechanisms or in general DNA metabolism, the focus here is on their functions in relation to telomeric DNA replication. When replication machinery unwinds duplex of telomeric DNA, G-quadruplex (G4) DNA structure can be formed on the G-rich strand of telomeres, which is basically used as a template of lagging strand synthesis. Werner syndrome RecQ-like helicase (WRN), Bloom syndrome RecQ-like helicase (BLM), and regulator of telomere elongation helicase 1 (RTEL1) resolve G4 DNAs in concert with single-stranded DNA binding proteins. These helicases also participate in resolution of D-loop (displacement loop) at the base of T-loop (telomere loop) structure. Disassembly of T-loop is required for replication fork to arrive at the end of chromosome. In the absence of RTEL1, persistent T-loop will be one of substrates of structure-specific SLX4-associated endonucleases. Structural barriers to replication fork are also generated by R-loop derived from telomeric repeat-containing RNA (TERRA) binding to telomeric DNA, and by topological stress along chromosome. Furthermore, homologous recombination of the telomeric DNA should be tightly regulated, because inappropriate recombination causes telomere defects such as multi-telomeric signals, sister telomere association, and end-to-end fusion of chromosomes. Proper recombination at the stalled replication fork is also essential for stability and restart of the fork. See the main text for details.

Eukaryotic genome integrity is maintained by protecting telomeres from various problems caused by their terminal position. Incomplete lagging-strand synthesis at the chromosomal termini causes gradual loss of genetic information. The iterative telomerase action or a homologous recombination-mediated mechanism, called Alternative Lengthening of Telomeres (ALT), is therefore needed to extend and maintain the repetitive TTAGGG sequences [14,103]. Moreover, the protective shelterin complex prevents chromosomal fusions resulting from improper activation of DNA repair pathways [1,2,3]. These mechanisms are essential for genomic stability, but at the same time they cause difficulties in replication. Telomeric repeats impede the replication machinery not only in telomeres, but also in interstitial chromosomal regions that contain the repeats, or when transferred to plasmid DNAs [48,55,104,105,106], suggesting that the replication difficulties can, at least partly, be attributed to the telomeric sequences themselves. Repetitive TTAGGG sequences can form G4 structures that are more stable than the standard B-form DNA duplex, thereby presenting obstacles to the progression of replication forks [9,107] (Figure 2a). Furthermore, G4-independent fork stalling on telomeric G-rich templates has been suggested by the results of in vitro experiments [108]. In addition, protective capping structures formed by shelterin can cause replication impediments (Figure 2a). T-loop structures, as well as telomeric R-loops, DNA topological constraint, and heterochromatin may interfere with replication fork progression if they are not resolved (Figure 2a). Therefore, a number of accessory proteins are required for efficient passage of replication forks through telomeres. Whereas shelterin proteins are potential obstacles to conventional replication forks, because they bind tightly to telomeric chromatin [104,109], evidence now indicates that shelterin components facilitate replication by recruiting additional proteins that resolve other obstacles (Figure 2b). Here, we provide an update of the mechanisms that are known to underlie efficient fork progression through telomeres.

### 3.2. TRF1 and RecQ-Like Helicases

TRF1, a shelterin component that is not essential for telomere capping, contributes to efficient replication in mammalian telomeres [48,55,56,110]. TRF1 deletion leads to various telomeric defects, including the fragile telomere phenotype, in which FISH (fluorescence in situ hybridization) signals of telomeric probes show multiple foci at single chromosomal termini on metaphase spreads. Although detailed mechanisms of this phenotype remains to be clarified, the multi telomeric signals are thought to be a consequence of replication defects at telomeres and to reflect telomeric DNA breakage or the presence of aberrant, condensed structures. Fragile telomeres are also observed with replication stress induced by low doses of aphidicolin, an inhibitor of DNA polymerases [48,55]. Furthermore, TRF1-deleted cells exhibit activation of the DNA-replication-checkpoint kinase ATR, sister telomere association, and ultrafine anaphase bridges in mitosis, which is consistent with the presence of replication defects [48,55,111,112].

One of the suggested molecular mechanisms for the suppression of fragile telomeres by TRF1 is the recruitment of Bloom syndrome protein (BLM) [48,56], a member of the RecQ-like (RECQL) helicase family [113] that can resolve G4 DNA, D-loop (displacement loop) structures, and Holliday-junction DNA in vitro [114,115,116,117,118]. During DNA replication, G4-forming ssDNA can be produced at telomeres by unwinding of duplex DNA or unfolding of the G-overhang in the T-loop [9]. Although ssDNA-binding proteins such as RPA and POT1 can counteract G4 formation [119,120,121,122,123,124], a single-DNA-molecule-based analysis revealed that deletion of BLM decreases the rate of progression of replication forks inside telomeric tracts, and a G4-stabilizing agent enhances this slowing down of the forks, supporting the idea that BLM promotes telomeric replication by resolving G4 DNA [46]. Indeed, BLM deficiency induces fragile telomere specifically in daughter chromatids derived from G-rich templates, but not from C-rich ones [48,56,58]. In addition to the resolution of G4 during S phase, BLM localizes to telomeres in G2/M and is involved in the processing of late- or post-replicative telomeric structures resulting from both leading- and lagging-strand replication, as well as in T-loop resolution [58,125,126]. BLM acts on ultrafine anaphase bridges, a subset of which originate from telomeric DNA, to resolve these aberrant post-replicative structures that might arise from incomplete replication [58,127]. BLM can bind to basic patches in the hinge domain of TRF1, and a TRF1 variant lacking the BLM-binding patches is defective in the suppression of fragile telomeres in TRF1-deleted cells [56]. Although TRF1 has been suggested to be the major factor in the recruitment of BLM to telomeres, the helicase activity of BLM can be modulated by other shelterin components, such as TRF2 and POT1 [128,129,130].

Similar to BLM, the RECQL Werner syndrome helicase (WRN) has been implicated in resolution of telomeric G4 DNA, D-loops, and Holliday junctions, and its activity is regulated by several shelterin components [114,116,128,129,131,132,133]. The helicase activity of WRN is required for efficient replication of G-rich telomeric DNA, and its deficiency causes loss of the telomeres that use the G-rich strand as a template for synthesis [58,134,135]. Stabilization of G4 DNA perturbs telomere replication and enhances association of WRN and BLM with telomeres [136]. However, unlike BLM, deficiency of WRN does not induce the multi-telomeric signals indicative of fragile telomeres [48]. WRN is thought to be recruited by TRF2 to telomeres in S phase, and is also involved in the control of telomeric recombination events, such as T-loop assembly and disassembly, repression of sister chromatid exchange, and ALT [128,131,135,137,138,139,140]. Overall, WRN and BLM seem to have partially shared but non-redundant functions for the common goal that is complete replication of the chromosome ends.

RECQL helicase 4 (RECQL4) is altered in patients with Rothmund–Thomson syndrome, and cells derived from these patients show telomere fragility [141]. The N-terminal non-catalytic region of RECQL4 has an essential role in the initiation of DNA replication, and is a metazoan homolog of yeast Sld2 (Drc1) [142,143]. RECQL4 localizes to telomeres in S phase, and knockdown of RECQL4 causes telomere dysfunction-induced foci (TIFs) and fragile telomeres. In contrast to BLM and WRN, RECQL4 does not possess G4-unwinding activity in vitro [144], although the N-terminal region binds to G4 structures [145]. Interaction of another RECQL protein, RECQL1, with TRF2 and flap endonuclease 1 (FEN1) has also been proposed to participate in telomere replication [146,147]. In vitro, RECQL1 can resolve D-loops and Holliday junctions, but not G4 DNA, and it displaces TRF1 and TRF2 from telomeric repeats [146,148,149]. However, the detailed molecular mechanisms for how these RECQL helicases maintain telomere integrity during replication are not yet known.

### 3.3. RTEL1

RTEL1 is a G4-resolving helicase that is involved in telomeric DNA replication [81,150]. RTEL1-knockout mouse embryonic fibroblasts have various chromosomal abnormalities, such as fragile telomeres, telomere circles (extrachromosomal circular DNAs that contain telomeric repeat sequences), and loss of telomere signals [48,57,151,152,153]. RTEL1 contains a PIP (proliferating cell nuclear antigen (PCNA)-interacting protein) box in its C-terminal region [153]. PCNA is a fundamental component of the replication machinery that increases the processivity of DNA polymerases. The PIP box of RTEL1 is required for unwinding of G4 DNAs not only in telomeres but also genome-wide during replication [153]. A PIP box-deleted variant of mouse RTEL1, which is defective in PCNA interaction, fails to rescue the fragile telomere phenotype induced by RTEL1 deletion, but can rescue telomere circles and telomere loss [153], suggesting that RTEL1 has at least two distinct and separable functions for telomere maintenance.

The T-loop structure is essential to protect chromosome ends, but this structure must be unwound and reformed during telomere replication. RTEL1 has been proposed to be a helicase that unwinds the T-loop, in which G-overhang DNA invades the double-stranded telomere [57,151,152]. In vitro, RTEL1 preferentially unwinds a 3′-ssDNA-invaded D-loop (which resembles the structure in the T-loop) in a RPA-dependent manner [154]. Telomere-circle formation and telomere loss in RTEL1-deficient cells support the idea that RTEL1 has a role in T-loop disassembly in vivo [57]. TRF2 is a binding partner of RTEL1 [70], and they interact via the TRFH dimerization domain of TRF2. A mutation that affects the TRFH domain and disrupts the TRF2-RTEL1 interaction leads to telomere-circle formation and telomere loss [70]. In patients with Hoyeraal–Hreidarsson syndrome (a severe variant of dyskeratosis congenita), mutation affects the RTEL1 C4C4 metal-binding motif [150], so that RTEL1 no longer binds to TRF2, and this RTEL1 variant fails to rescue the telomere loss and the telomere circles induced by RTEL1 deletion [70]. Because the C4C4-defective RTEL1 variant can rescue the fragile telomere phenotype, the interaction of RTEL1 with TRF2 seems to be required for proper disassembly of the T-loop, rather than G4-unwinding, preventing loss of the telomere as a circle. Taken together, RTEL1 prevents telomere fragility via interaction with PCNA and facilitates T-loop disassembly via interaction with TRF2. However, TRF2 is also essential for the assembly of the T-loop [7,8,155]. How these contrasting activities of TRF2 are regulated during the cell cycle is not currently known.

### 3.4. SLX4

Telomere-circle formation and telomere loss in RTEL1-deficient cells are mediated by SLX4 (also known as FANCP or BTBD12), which serves as a scaffold protein for the structure-specific endonucleases SLX1, XPF, and MUS81 [57,156,157,158,159]. The SLX4–endonuclease complex is capable of nucleolytically resolving D-loops and Holliday junctions in vitro [71,126,156,157,158], and is involved in genome-wide resolution of Holliday junctions, and in repair of interstrand DNA cross-links [160,161,162,163]. Deletion of SLX4, SLX1, or XPF, but not MUS81, suppresses telomere-circle formation that is observed in the absence of RTEL1 [57], suggesting that SLX4–endonuclease complexes excise persistent T-loop structures. Furthermore, deletion of SLX4 leads to TIFs and fragile telomeres [72,126,164], suggesting that SLX4-mediated nucleolytic resolution of branched intermediates is required during telomere replication.

In human cells, SLX4 localizes to telomeres throughout the cell cycle via binding to the TRF2 TRFH domain [71,72]. Although SLX4 in mice is involved in telomere maintenance [57,72], the TRF2-binding motif of SLX4 (HxLxP) is conserved in primates, but not in non-primate mammals. The Holliday junction-processing activity of human SLX4 is carefully regulated by TRF1, TRF2, and BLM, preventing inappropriate telomere shortening by T-loop excision and aberrant crossover between telomeric sister chromatids [71,126,164,165]. Recently, SUMO was shown to regulate the function of human SLX4, including TRF2 binding [166,167,168], further contributing to the tight regulation of SLX4 activity for homeostasis of telomere length.

### 3.5. FEN1 and DNA2

FEN1, a structure-specific endonuclease, is important for proper telomere replication, independent of its general role in Okazaki fragment maturation. FEN1 has been suggested to facilitate telomeric replication by reinitiating stalled replication forks [169,170]. FEN1 depletion leads to a fragile telomere phenotype and to loss of single sister telomeres derived from lagging- or leading-strand replication [169,170,171]. Nuclease activity and interaction with WRN and TRF2 are required for FEN1 to prevent telomere fragility [169,170,171]. Although FEN1 cleaves telomeric G4-containing 5′ flaps in vitro [172], in vivo substrates during telomere replication are unknown [173]. Notably, RNase H1, an endoribonuclease that degrades the RNA strand of a DNA–RNA hybrid, can rescue the telomeric replication defect in FEN1-deleted cells [171].

DNA2, a multifunctional 5′–3′ DNA helicase with exonuclease and endonuclease activities, participates in Okazaki fragment maturation and processing of G4 DNA [173]. DNA2 heterozygous knockout in mice causes fragile telomere phenotype and telomere loss without genome-wide effects on replication [174], although the mechanisms for DNA2 function at telomeres remain to be determined.

### 3.6. UPF1 and Chromatin Remodelers

The up-frameshift suppressor 1 (UPF1, also known as RENT1 or SMG2) is a DNA/RNA-dependent ATPase and 5′–3′ helicase, known as a component of the RNA quality control machinery [175,176]. UPF1 ATPase activity is required to prevent telomere dysfunctions during replication [177]. UPF1 binds to telomeres through interaction with the shelterin component TPP1 [84,178]. UPF1 knockdown results in DNA damage at telomeres and frequent loss of the telomeres that are replicated by leading-strand synthesis [178]. UPF1 knockdown also results in an increased level of TERRA signal at telomeres, suggesting a role for UPF1 in displacement of TERRA [84,178]. If it is not displaced, TERRA can form a telomeric R-loop by binding to the C-rich DNA strand, and might induce replication stress and double-strand breaks during leading-strand DNA replication. Furthermore, the chromatin remodeling protein ATRX has been implicated in the displacement of TERRA to resolve recombinogenic DNA–RNA hybrid structures [179,180]. Loss of ATRX is associated with ALT in cancer cells, in which RNase H1 regulates TERRA–telomeric-DNA hybrids and telomere maintenance [179,181,182,183,184]. Deletion of mouse INO80, encoding a chromatin remodeler involved in diverse aspects of DNA metabolism [185], also results in fragile telomere phenotype [186].

### 3.7. Apollo

Apollo (also known as SNM1B) is a member of the metallo-β-lactamase/β-CASP family, and has 5′–3′ exonuclease activity [187]. Apollo has been implicated in several DNA damage responses including ATM activation and Fanconi anemia pathway [188,189,190,191,192]. Besides these genome-wide functions, Apollo has telomere-associated functions. Evidence indicates that Apollo localizes to telomeres through its interaction with TRF2 [67,193,194,195,196]. Studies of crystal structures showed that the C-terminal YxLxP motif of Apollo is involved in binding to TRF2, which requires the F120 residue of the TRF2 TRFH domain [67]. Knockdown of DCLRE1B, which encodes human Apollo protein, results in fragile telomere phenotype in telomeres produced by both lagging- or leading-strand replication [195]. Expressions of Apollo mutant proteins lacking the TRF2-binding or nuclease activity have dominant-negative effects on telomeric DNA replication [197,198]. Apollo has been suggested to act in the same pathway as DNA topoisomerase 2α (TOP2A), which relieves accumulating topological stress during human telomere replication [197]. However, exactly how the exonuclease activity of Apollo contributes to telomere replication is unknown. On the other hand, mouse TOP2A is recruited to telomeres in a manner that is dependent on TRF1, and which prevents the fragile telomere phenotype [112].

Mouse Apollo has a further essential role in the generation of the telomeric G-overhang after the bulk replication of telomeres [193,199,200]. Because leading-strand replication on the C-rich strand generates a blunt-ended daughter telomere, 5′-end resection of the C-rich strand by Apollo is required to form the single-stranded G-overhang. DCLRE1B-knockout mouse embryonic fibroblasts exhibit TIFs, especially in S phase, and have leading-end telomere fusion [193,199,200]. Such role of human Apollo remains to be clarified. Additional aspects of G-overhang generation, such as repression of Apollo by POT1 and following resection by exonuclease 1 (EXO1), are reviewed elsewhere [2,4,14].

### 3.8. POT1 and RAP1

POT1 is a shelterin component with multiple functions, and it binds directly to telomeric ssDNA. A well-documented role of POT1 is to protect the G-overhang from DNA repair activities by excluding RPA and ATR activation from the 3’ overhang ssDNA [201,202,203,204,205,206]. Other functions of POT1 in the regulation of G-overhang generation and telomere length (by controlling telomerase activity) are reviewed in detail elsewhere [103,207,208]. Moreover, POT1 has been proposed to overcome RPA accumulation on ssDNA during DNA replication and to repress sister telomere association [56,134]. TRF1 acts as a platform to recruit POT1 through the interaction mediated by TIN2–TPP1 in shelterin [56,205]. Mutations encoding POT1 variants defective in ssDNA binding have been found in patients with cancer, and expression of these variants elicits fragile telomere and ATR-dependent TIFs, which are signs of telomeric replication defects [209,210,211,212]. The function of POT1 in efficient replication seems to be mediated by the interaction with the CST (CTC1–STN1–TEN1) ternary complex [212], which stimulates replication fork restart. Knockdown of CTC1 or STN1 induces fragile telomere and TIFs and is epistatic to POT1 mutations [212]. Another function of POT1 is the unwinding of G4 DNA on the G-rich template strands [121,122,123,124]. In parallel, mouse Rap1, a shelterin component that interacts with TRF2, is required to prevent telomere fragility, telomere recombination, and telomere shortening, whereas human RAP1 inhibits chromosome fusions at telomeres [213,214,215].

### 3.9. The CST Complex

The CST complex is a ssDNA-binding complex, which is structurally related to RPA, and which is involved in the regulation of telomeric G-overhangs [216,217,218]. Besides high-affinity binding to telomeric ssDNA, interaction with TPP1–POT1 heterodimer regulates the telomeric localization of the CST complex [200,219,220]. CST stimulates RNA priming and DNA synthesis by the primase-Polα complex to fill in the C-strand after G-strand extension by telomerase and/or EXO1-mediated resection [200,220,221,222,223,224,225,226]. Because replication forks stall naturally at mammalian telomeres, an ATR-dependent fork restart mechanism is needed to complete DNA replication [227,228,229]. Knockdown of expression of CST components decreases bromodeoxyuridine incorporation at telomeres after release from hydroxyurea-induced replication fork arrest, and elicits telomere fragility [212,226,230,231,232,233,234,235,236]. Several lines of evidence suggest that CST contributes to fork restart not only in telomeres but also genome-wide under conditions of replication stress [232,233,235,237]. Stimulation of the primase-Polα complex has been implicated in the restart of stalled fork [226,231,236], but DNA-fiber analysis has also suggested that CST promotes replication recovery partly by activating dormant origins [232,235,237]. Results of deep-sequencing analysis revealed that CST recruits RAD51, a recombination protein, to GC-rich repetitive regions including telomeres in response to hydroxyurea [238]. The DNA repair protein BRCA2 also contributes to telomere replication as a RAD51 loader [239]. Recruited RAD51 would facilitate fork restart by strand exchange of the collapsed fork.

## 4. Restart of Replication to Complete Telomere Duplication

**Figure 3:**
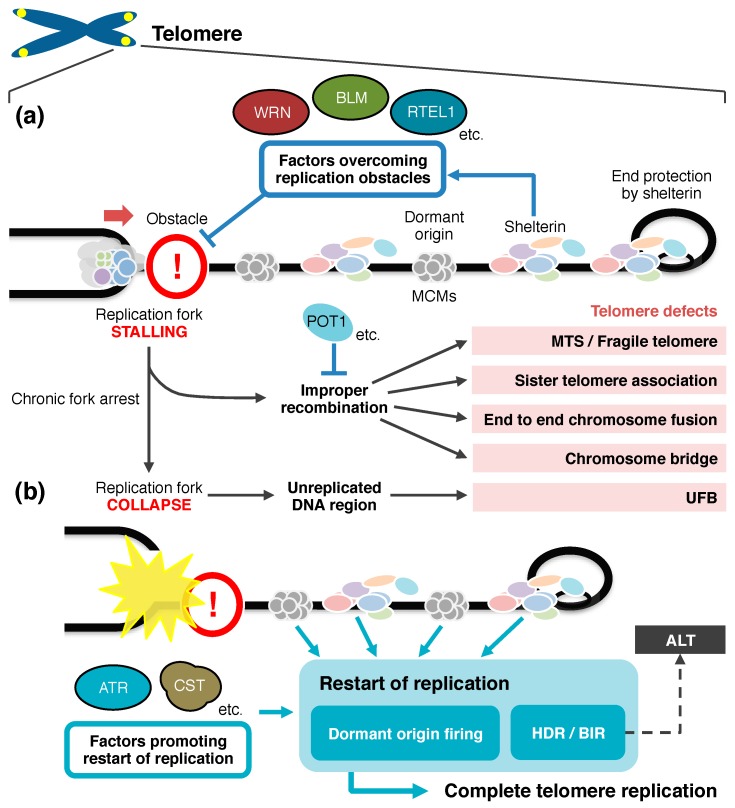
A model for the consequences of telomeric replication fork arrest and the different recovery mechanisms. (**a**) Telomeres present intrinsic obstacles, impeding the passage of replication machinery. Shelterin and accessory proteins prevent the fork stalling and repress improper recombination activity. Inappropriate recombination of the telomeric fork may result in telomere defects. (**b**) Persistent fork arrest might lead to fork collapse at telomeres. Restart of replication is necessary to avoid leaving an unreplicated region. Dormant origins could fire to complete telomere replication. Homologous recombination is also a general recovery mechanism from replication fork collapse. However, recombination at telomeres might increase the risk of cellular immortalization by ALT (alternative lengthening of telomeres). It is currently unknown how shelterin exactly contributes to restart of replication. BIR: break-induced replication; HDR: homology-directed repair; MTS: multi-telomeric signals; UFB: ultrafine anaphase bridge.

Prolonged replication fork arrest ultimately leads to irreversible fork collapse (Figure 3) [240]. In general, broken forks are rescued by incoming replication forks or repaired by recombination-mediated fork-restart mechanisms [241,242]. If no dormant origin exists in the telomeric region distal to the broken fork, the region may remain unreplicated. Therefore, loading of backup MCM replicative helicase in the telomere may be particularly important for the completion of telomere duplication. Homologous recombination-mediated processes, such as break-induced replication, provide alternative pathways to rescue the collapsed replication fork [243,244]. Recently, break-induced replication by PCNA–Polδ was shown to occur at mammalian telomeres [245,246]. MCM helicase may be dispensable for the break-induced telomere synthesis, and the DNA-unwinding mechanism in this process is unknown [246]. It has been suggested that break-induced replication promotes ALT to maintain the telomere length in telomerase-negative cancer cells [245,246]. The rescue of fork collapse by firing of dormant origins may contribute to prevent such aberrant telomere extension.

## 5. Concluding Remarks

Efficient replication of telomeric DNA requires a number of interactions between telomere-specific proteins and non-telomere-specific proteins to support fork progression (Figure 2). In the absence of these factors, replication forks frequently stall, collapse, and give rise to aberrant recombination, leading to telomere fragility. Unreplicated regions of telomeres or improper recombination such as sister telomere association may cause aberrant chromosome segregation in mitosis (Figure 3). Because the factors that overcome the impediments to telomeric replication are also involved in general DNA replication, repair, and recombination, and are sometimes essential for viability, separation-of-function mutants have been valuable tools to elucidate the mechanisms for the preservation of telomere integrity. Telomeres regulate cellular lifespan and their dysfunction is a driver of genomic instability. Besides the simple telomere protection, efficient replication of telomeres has emerged as another factor that influences aging and carcinogenesis [55,150,212].

It is now clear that DNA replication at telomeres is supported by multiple mechanisms, which are discussed in this review and another recent review [247]. However, much remains unknown about how these mechanisms are controlled during the cell cycle, differentiation, aging, and cancer development. In particular, several factors appear to have opposing effects on telomeric DNA replication. For example, G4 DNA may contribute to specification of replication origins, but impairs replication fork progression. Activation of the ATR-dependent checkpoint pathway is repressed by POT1 at telomeres, but ATR is required to prevent telomere fragility. In addition, although R-loops formed by TERRA transcripts are an obstacle to the replication machinery, TERRA is suggested to promote the switch from RPA to POT1 at the G-overhang after replication [10,11]. How are the apparently conflicting roles of these factors coordinated? One major future challenge is to understand how telomeres manage to complete their duplication while avoiding the potential harmful effects of this process, including replication stress, telomere shortening, and genomic instability. Whether the replication machinery itself modulates a complex network of telomeric protein–protein interactions in response to fork stalling is an important question. Indeed, Timeless, a component of the fork protection complex that travels with the replication fork, is required for efficient telomere replication, and interacts with TRF1 and TRF2 [248]. Posttranslational modifications of telomeric factors and nuclear localization of telomeres might determine the appropriate use of multiple factors at telomeres.

Further elucidation of the molecular mechanisms that ensure efficient telomere replication is an important issue in telomere biology. The molecular mechanisms that coordinate dormant origin firing, homology-directed repair, and break-induced replication in response to fork collapse at telomeres are largely unknown. Another question is whether telomere length has an impact on telomere fragility. It is well known that short telomeres cause telomere deprotection and cell death. On the other hand, longer telomeres might increase the probability of fork stalling and collapse, leading to telomere loss. Comparing the frequency of telomeric fork stalling, collapse, or restart in broad biological contexts (e.g., normal vs. cancer cells, young vs. old cells) could provide insights into the endogenous sources of telomere fragility. Furthermore, it is important to uncover the molecular mechanisms underlying abnormal telomere shortening and cancer predisposition in short telomere diseases (so-called telomeropathy), such as Werner syndrome and Hoyeraal–Hreidarsson syndrome [150,198], which are caused by mutations in genes encoding factors involved in telomere replication. It will be critical to understand how semi-conservative replication influences telomere elongation by telomerase and vice versa. A comprehensive and integrated understanding of these processes could yield novel targets and strategies for disease diagnosis and therapy.
